# Global analysis of dorsoventral patterning in the wasp *Nasonia* reveals extensive incorporation of novelty in a regulatory network

**DOI:** 10.1186/s12915-016-0285-y

**Published:** 2016-08-01

**Authors:** Daniel Pers, Thomas Buchta, Orhan Özüak, Selma Wolff, Jessica M. Pietsch, Mohammad Bilal Memon, Siegfried Roth, Jeremy A. Lynch

**Affiliations:** 1 Department of Biological Sciences, University of Illinois at Chicago, MBRB 4020, 900 S. Ashland Avenue, Chicago, IL 60402 USA; 2 Institute for Developmental Biology, University at Cologne, Cologne, Germany

## Abstract

**Background:**

Gene regulatory networks (GRNs) underlie developmental patterning and morphogenetic processes, and changes in the interactions within the underlying GRNs are a major driver of evolutionary processes. In order to make meaningful comparisons that can provide significant insights into the evolution of regulatory networks, homologous networks from multiple taxa must be deeply characterized. One of the most thoroughly characterized GRNs is the dorsoventral (DV) patterning system of the *Drosophila melanogaster* embryo. We have developed the wasp *Nasonia* as a comparative DV patterning model because it has shown the convergent evolution of a mode of early embryonic patterning very similar to that of the fly, and it is of interest to know whether the similarity at the gross level also extends to the molecular level.

**Results:**

We used RNAi to dorsalize and ventralize *Nasonia* embryos, RNAseq to quantify transcriptome-wide expression levels, and differential expression analysis to identify genes whose expression levels change in either RNAi case. This led to the identification of >100 genes differentially expressed and regulated along the DV axis. Only a handful of these genes are shared DV components in both fly and wasp. Many of those unique to *Nasonia* are cytoskeletal and adhesion molecules, which may be related to the divergent cell and tissue behavior observed at gastrulation. In addition, many transcription factors and signaling components are only DV regulated in *Nasonia*, likely reflecting the divergent upstream patterning mechanisms involved in producing the conserved pattern of cell fates observed at gastrulation. Finally, several genes that lack *Drosophila* orthologs show robust and distinct expression patterns. These include genes with vertebrate homologs that have been lost in the fly lineage, genes that are found only among Hymenoptera, and several genes that entered the *Nasonia* genome through lateral transfer from endosymbiotic bacteria.

**Conclusions:**

Altogether, our results provide insights into how GRNs respond to new functional demands and how they can incorporate novel components.

**Electronic supplementary material:**

The online version of this article (doi:10.1186/s12915-016-0285-y) contains supplementary material, which is available to authorized users.

## Background

Patterning and morphogenetic processes in developmental systems rely on the underlying activity of gene regulatory networks (GRNs) [1]. Changes in these networks can lead to new developmental outputs (morphologies, cell types) and thus understanding how these networks vary across phylogenies is critical to understanding the evolution of development [2].

To understand evolutionary variation in GRNs, a comparative approach must be taken. In addition, the networks to be compared must be understood at a high level of detail and completeness if the comparisons are to be robust and valuable sources of evolutionary insight [3]. The embryonic dorsoventral (DV) patterning network of *Drosophila melanogaster* is one of the few GRNs that are understood well enough to serve as a basis for comparative analysis.

DV patterning in *Drosophila* leads to the establishment of three broad cell fates, the mesoderm, ectoderm, and the amnioserosa, with distinct sub-fates established within each (particularly the ectoderm) [4]. The NF-kB transcription factor Dorsal is a master regulator of this network, and acts as a morphogen, activating and repressing genes in a concentration-dependent manner [5, 6]. Dorsal itself has direct regulatory input into most of the components of the *Drosophila* DV GRN [7], and its patterning ability is augmented by additional regulatory interactions among its targets that lead to refinement of patterning (e.g., [4, 8–10]). Feedback on Toll signaling by one of its zygotic targets has recently been demonstrated [11]. The function of this feedback appears to be to stabilize the breadth and shape of the Dorsal gradient in the face of fluctuating and imprecise upstream positional information, allowing Dorsal to most efficiently perform its function at the top of the DV patterning hierarchy [12].

In contrast to *Drosophila,* patterning processes that are dynamic in both space and time, and are generated by regulatory networks with apparent self-regulatory properties, have been found in other insect species [13–16]. In order to understand how early embryonic patterning networks can be altered in the course of evolution, we have endeavored to characterize the embryonic DV GRN of the wasp *Nasonia* at a level of detail that makes meaningful comparisons to *Drosophila* possible. *Nasonia* and *Drosophila* have been evolving independently for over 300 million years [17], yet they undergo very similar modes of long germ embryogenesis, which have likely arisen through convergent evolution [18].

**Fig. 1:**
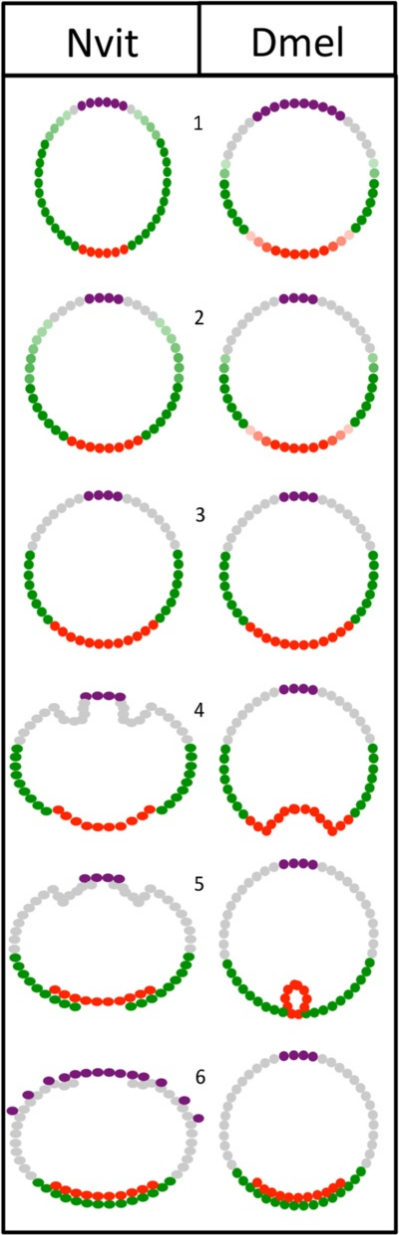
Summary of similarities and differences between *Nasonia* and *Drosophila* embryogenesis. Representation of wasp (*left*) and fly (*right*) embryos from the mid-blastoderm stage (*1*) through the completion of gastrulation (*6*). Patterning and tissue establishment is represented by the following colors: extraembryonic (*purple*), dorsal ectoderm (*gray*), neuroectoderm (*green*), mesoderm (*red*). As the blastoderm cellularizes (*1–3*), retraction of the expression domain of extraembryonic marker genes is observed in both insects. While the domains of the other tissue-specific markers fluctuate very little in the fly, the mesoderm marker gene expression domain expands dynamically at the expense of lateral ectoderm marker expression in the wasp. These wasp-specific changes precede a stable pattern nearly identical to that of the fly just prior to the start of gastrulation (*3*). However, once gastrulation begins (*4–6*), the behavior of the different tissue domains diverges again. In the fly a ventral furrow internalizes the mesoderm. In the wasp, the epithelium breaks at the border of the mesoderm and neuroectoderm, and the free edges of the ectoderm migrate towards each other until they meet and re-form a continuous epithelium at the ventral midline. At the end of gastrulation, the fly amnioserosa remains in place and slowly shrinks; however, the wasp extraembryonic tissue expands at this time. The dorsal ectoderm flanking the serosa and amnion folds and eventually breaks, again creating free edges. The free edges of the serosa then migrate over the ectodermal epithelium, forming a squamous epithelium that eventually covers the entire embryo. Dmel *Drosophila melanogaster*, Nvit *Nasonia vitripennis*

**Fig. 2:**
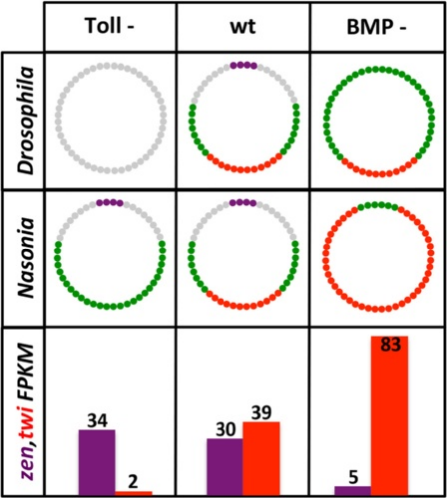
Summary of dorsoventral regulation and results of parental RNA interference (pRNAi) in *Nasonia*, compared to *Drosophila*. Extent of tissue domains in Toll mutant/RNAi (*Toll -*), wild type (*wt*), and BMP mutants/RNAi (*BMP -*) in *Drosophila* and *Nasonia*, illustrating that upstream patterning pathways have very different effects in these two species. Extraembryonic (*purple*), dorsal ectoderm (*gray*), neuroectoderm (*green*), and mesoderm (*red*) are shown. The lower three boxes show relative, normalized expression levels (given in fragments per kilobase of transcript per million mapped reads, *FPKM*) for *Nv-zen* (marker of extraembryonic tissues) and *Nv-twist* (marker of mesoderm tissue) in Toll -, wt, and BMP -

The expression of marker genes for the major tissue types along the DV axis (mesoderm, ectoderm, and extraembryonic membranes) are nearly identical at the onset of gastrulation in the two species (Fig. 1; [13]). However, the ways these patterns are generated are quite divergent, as the *Nasonia* DV patterning system exhibits dynamic behavior, with initially narrow domains of ventral genes expanding over developmental time, indicating the possibility that a positive feedback loop is used to generate the full DV pattern in *Nasonia* (Fig. 1, [13]). In addition, it has been shown that generation of the DV pattern in *Nasonia* depends heavily on BMP signaling, with Toll signaling relegated to a limited role on the ventral side (Fig. 2, [14]). This is in direct contrast to the *Drosophila* system, where Toll signaling is responsible for all DV polarity, and BMP plays a subordinate, dorsally restricted role (Fig. 2, [19]).

The *Nasonia* embryo diverges from *Drosophila* again at the onset of gastrulation (Fig. 1). There is no ventral furrow in *Nasonia* gastrulation, but rather the blastodermal epithelium ruptures at the border between mesoderm and mesectoderm, and the ectoderm then appears to crawl over the mesoderm until the two ectodermal plates meet at the ventral midline [13]. In addition, *Nasonia* has a true serosa, which migrates dramatically, unlike the fly amnioserosa, which remains on the dorsal side until it disappears at dorsal closure (Fig. 1, [13, 20]). Both of these *Nasonia* morphogenetic processes are similar to those in *Apis*, indicating that they were an innovation of the hyper-diverse higher Hymenoptera (Apocrita) [21].

These differences indicate that the composition of, and regulatory connections within, the *Nasonia* DV patterning network are significantly different from their counterparts in *Drosophila*, despite the near identity of the pattern of tissue fate marker expression at the end of the blastoderm stage. The availability of both RNAi to manipulate gene function and well-characterized genomic and transcriptomic data make *Nasonia* an ideal model for comparative GRN analysis [22]. We have previously shown that RNAi knockdown of BMP signaling components leads to a robust loss of dorsally and laterally expressed genes along with a simultaneous expansion of some ventrally expressed mesoderm markers (Fig. 2, [14]). On the other hand, reduction of Toll signaling leads to the loss of the mesoderm and ventral ectoderm (Fig. 2). We reasoned that comparing the global transcriptomes of these two RNAi cases to each other and to wild type (wt) should allow us to discover most genes that are differentially expressed along the DV axis of the wasp.

Using this approach, we have identified 110 genes with distinct expression patterns in the *Nasonia* embryo. While there is core of conserved DV factors found in both *Nasonia* and *Drosophila*, a significant majority of *Drosophila* orthologs of *Nasonia* DV patterned genes do not show DV expression patterns in the fly embryo. Other highlights include a set of factors that are differentially expressed along the DV axis in both species, but mark different cell fates in *Nasonia*; *Nasonia* genes that do not have clear homologs in *Drosophila* (or in any other animal in many cases); and new expression patterns that do not have clear equivalents in the *Drosophila* embryo. This study represents the first major leap towards a comprehensive characterization of an additional DV patterning GRN for comparative analysis of regulatory network evolution.

## Materials and methods

### Experimental design

 As the focus of our transcriptome analysis, we used embryos of the parasitoid wasp *Nasonia vitripennis* at the time period starting from the penultimate syncytial division until the onset of gastrulation (4–7 hours after egg lay at 28 °C). This covers the dynamic patterning stage until the final establishment of cell fates on the blastoderm and onset of gastrulation, and represents the critical patterning time window in *Nasonia*. Total RNA was collected from 4–7-h-old *Nv-TollA*, *Nv-dpp,* and *Nv-gbb* parental RNA interference (pRNAi) embryos, as well as from mock-injected embryos of the same age. For each pool, embryos were collected over the period of 1 week, and the number of embryos in each pool was estimated to be greater than 1000. For *Nv-TollA*, two independent double-stranded RNAs (dsRNAs) made from non-overlapping sections of the Toll gene were used in separate experiments, serving as biological replicates. *Nv-dpp* and *Nv-gbb* served as biological replicates for BMP signaling knockdown, as these two genes produce indistinguishably ventralized progeny. Each dsRNA was at a concentration of 1–2 μg/mL in water. Finally, two independent water-injected embryo pools were collected. RNA was isolated from embryos using standard Trizol-based protocols.

### Sequencing

Libraries were prepared using the Illumina® TruSeq® RNA Sample Preparation Kit. Library preparation started with 2 μg of total RNA. After poly-A selection (using poly-T oligo-attached magnetic beads), mRNA was purified and fragmented using divalent cations under elevated temperature. The RNA fragments underwent reverse transcription using random primers. This was followed by second strand cDNA synthesis with DNA Polymerase I and RNase H. After end repair and A-tailing, indexing adapters were ligated. The products were then purified and amplified (14 PCR cycles) to create the final cDNA libraries. After library validation and quantification, equimolar amounts of library were pooled. The pool was quantified using the Peqlab KAPA Library Quantification Kit and the Applied Biosystems 7900HT Sequence Detection System. The pool was sequenced using an Illumina TruSeq PE Cluster Kit v3 and an Illumina TruSeq SBS Kit v3-HS on an Illumina HiSeq 2000 sequencer with a paired-end (101 × 7 × 101 cycle) protocol.

### Analysis

The quality of the resulting sequences was checked using fastQC and the sequences were then processed for entry into the Cufflinks package. The procedure outlined in [23] was followed with slight alterations, including updated software versions. Jobs sent to the CHEOPS cluster located at the University of Cologne contained the relevant parameters, and are presented in Additional file 1: Methods. We primarily relied on annotation 2.0 of the *N. vitripennis* genome, found at http://arthropods.eugenes.org/EvidentialGene/nasonia/genes/. This annotation was modified slightly to be compatible with the Cufflinks analyses. This altered file is available on request. Annotation 2.0 was mapped to version 1.0 of the *Nasonia* genome assembly (http://www.hymenopteragenome.org/drupal/sites/hymenopteragenome.org.nasonia/files/data/Nvit_1.0_scaffolds.fa.gz). Very similar but not identical results were obtained using annotation 1.2 combined with assembly 2.0: (http://www.hymenopteragenome.org/nasonia/?q=sequencing_and_analysis_consortium_datasets). Discrepancies are discussed as appropriate in the main text. The raw cuffdiff results for both experiments are provided as Additional files 2 and 3. A compilation of all genes that showed significant differential expression (DE) in one or more comparisons (regardless of whether our additional criteria were met) is presented in Additional file 4. Additional file 5 contains the annotations of the 110 genes with confirmed expression in this analysis.

Genes for expression analysis were chosen based on three factors: the q-value for significance of DE must be below 0.05, DE must be greater than 1.5× in at least one comparison, and the fragments per kilobase of transcript per million mapped reads (FPKM) of the gene must be above 3 in wt.

In situ hybridization was performed using standard protocols [13, 24].

### Comparison to *Drosophila*

*D. melanogaster* DV regulated genes were detected using the Annotation Search function on the Berkeley Drosophila Genome Project (BGDP) in situ homepage. Stage (stage 4–6) and Annotation Terms (mesoderm anlage *in statu nascendi*, amnioserosa anlage *in statu nascendi*, dorsal ectoderm anlage *in statu nascendi*, mesectoderm anlage *in statu nascendi*, ventral ectoderm anlage *in statu nascendi*) were used to refine search results. The resulting queries of CG numbers with hyperlinks to individual BDGP pages were then extracted and compiled into a database. Stage 4–6 in situ hybridization images for each CG were examined and annotated with an appropriate primary localization. The following localization terms were used: Extraembryonic (dorsal expression), Dorsal Ectoderm (dorsolateral localization), Ectoderm (lateral localization), Ventral Ectoderm (ventrolateral localization), Mesoderm (ventral midline localization), and Endoderm (anterior/posterior localization corresponding to mouth/anus precursors). An additional localization term, Complex, was used for genes with multiple localizations of equal expression. Genes not localized along the DV axis (Gap, Pair-rule, Segmental, ubiquitous, unlocalized, etc.) were removed from the database. Additionally, DV regulated genes detected by Stathopoulos et al. [25] were compiled into this database, resulting in a total of 278 genes. Each gene was then examined in greater detail by searching its CG number on flybase.org. Gene names, symbols, and molecular function were collected. Specific molecular functions referenced by flybase were then organized into the following broader molecular function categories: Membrane Proteins, Transcription Factors, Signaling, Catalytic Enzymes, Proteases, Kinases, Structural, Protein Binding, RNA Binding, DNA Binding, Ion Binding. Genes with no “Experimental Evidence” or “Predictions/Assertions” were categorized under the term Unknown. Potential *N. vitripennis* orthologs for each gene were found under the “Orthologs in non-Dipteran Insects” tab on each flybase page. NasviXXXXXXX numbers for each ortholog were extracted and added to the database. These Nasvi numbers were then used to search within the RNA-RNAseq data to determine if each ortholog was differentially expressed. If significant expression was found, the appropriate conditions were noted in the database. The results of this analysis are presented in Additional file 6.

## Results and discussion

**Fig. 3:**
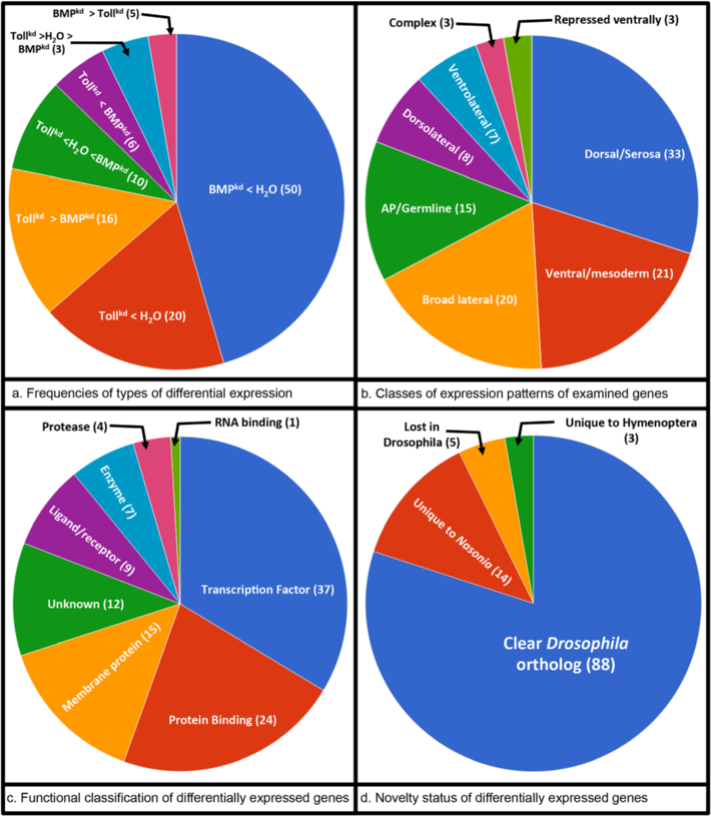
Summary of results of RNAi-RNAseq experiment. **a** General results of RNAi-RNAseq. The BMP^kd^ < H_2_O class consisted of genes showing decreased expression after BMP RNAi compared to water-injected embryos, and no significant difference after Toll RNAi. The Toll^kd^ < H_2_O class comprised genes showing decreased expression after Toll RNAi compared to control, and no significant difference after BMP RNAi. The Toll^kd^ > BMP^kd^ class consisted of genes in which differential expression was detected only when the Toll and BMP knockdowns were compared. The Toll^kd^ < H_2_O < BMPkd class contained genes that were, relative to control, significantly reduced in Toll RNAi and increased in BMP RNAi. The Toll^kd^ < BMP^kd^ class included genes that were only detected when Toll and BMP samples were compared, and where expression was reduced in Toll RNAi. The Toll^kd^ > H_2_O > BMP^kd^ class included genes that increased in Toll RNAi and decreased in BMP RNAi relative to water-injected embryos. The BMP^kd^ > Toll^kd^ class contained genes that were detected only when BMP and Toll expression levels were compared, with BMP RNAi expression levels being higher than Toll RNAi. **b** Enumeration of expression patterns of genes discovered in this analysis. **c** Enumeration of functional classes of genes discovered in this analysis. **d** Enumeration of homology status of genes uncovered in this analysis

The 110 genes identified in our RNAi-RNAseq analysis can be classified and categorized in several ways. Genes whose expression was significantly altered in at least one RNAi case fell into seven classes (Fig. 3a). Genes expressed all along the DV axis were detected, as well as some unexpected patterns at the anteroposterior (AP) poles and in the primordial germ cells (Fig. 3b). Many distinct molecular functions were present among the detected genes. Transcription factors were predominant, followed by membrane proteins, protein-binding proteins, and proteins of unknown function (Fig. 3c). Finally, while the majority of genes have clear *Drosophila* orthologs, 20 % do not (Fig. 3d). Some of these are Hymenoptera specific, whereas others are conserved within the vertebrates but have been lost in the fly lineage. These findings will be further described and discussed below. The initial description of the results is organized around the region of embryonic expression of the genes, because the nature of many of the patterns gives insights into how the *Nasonia* patterning system operates. Further categories of expression of molecule type also have distinct descriptions and discussion.

### Ventrally expressed genes give insights into the establishment and patterning of the mesoderm

**Fig. 4:**
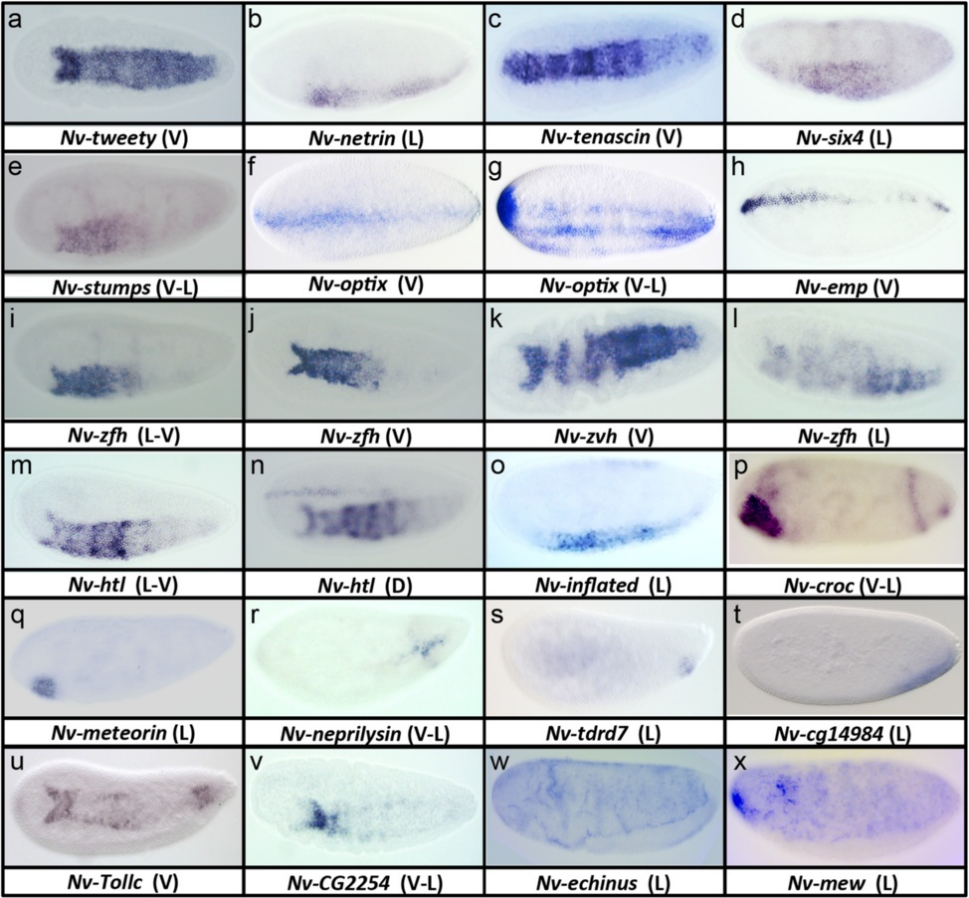
**a–x** Ventrally regulated genes in *Nasonia*. All embryos are in the last cell cycle of the syncytial blastoderm stage. *L* lateral view, *L-V* lateral-ventral view, *V* ventral view, *V-L* ventrolateral view

In all, we found 22 genes to be expressed in the presumptive mesoderm, or in a ventral region covering the ventral midline of the egg. All of these were significantly reduced after Toll pRNAi. Eight of the genes had increased expression after BMP knockdown, while 14 were not significantly changed after BMP knockdown. Genes in this latter class that are expressed in the full presumptive mesoderm include the *Nasonia* homolog of the calcium-responsive chloride channel *tweety* (Fig. 4a), the neural guidance ligand encoding gene *netrin* (Fig. 4b), and the adhesion molecule *tenascin-major* (Fig. 4c). The remaining 14 genes showed a significant increase in expression after BMP knockdown, similar to *Nv-twist* or *Nv-sna*. Thus, these genes are either directly repressed by BMP signaling, or are indirect BMP targets downstream of genes negatively regulated by BMP signaling. The expression patterns of *Nv-six4* (transcription factor, Fig. 4d) and *Nv-stumps* (FGF pathway component, Fig. 4e) support this hypothesis, as they only appear well after the initial *Nv-twi* domain initiates and, in the case of *Nv-six4*, just preceding the first movements of gastrulation.

Furthermore, we found no other genes that followed the pattern described for *Nv-twi* or *Nv-sna* [13], that is, an early narrow stripe of expression in nuclear cycles 10 and 11 that expands and changes to the mature “slug”-shaped domain. However, the expression of *Nv-optix* showed a variation on this pattern. An early, narrow ventral stripe was present at cycles 10 and 11, then expanded in cycle 12. Instead of filling a broad ventral domain, *Nv-optix* splits into three parallel stripes along the DV axis (Fig. 4f, g).

Only one gene was found to follow the expression dynamics of *Nv-cactus*, which is expressed early in a narrow ventral stripe and does not expand over time, but rather disappears at gastrulation, and does not expand after BMP knockdown [13, 14]. The single gene following this trend was *Nv-epithelial membrane protein* (*Nv-emp*), a homolog of a scavenger receptor (Fig. 4h). Like *Nv-cactus*, we found that *Nv-emp* was expressed in a narrow stripe that was not always complete along the length of the DV axis in cycle 10 and 11, and then was progressively lost in the main body of the embryo (while remaining at the poles) over the course of cycle 12. Scavenger receptors similar to *Nv-emp* (a homolog of CD36) have been shown to have a role in regulating NF-kB (the vertebrate homolog of Dorsal) activity in other systems [26], raising the intriguing possibility that we have uncovered a new Toll feedback control factor for embryonic patterning in *Nasonia.* Further, this gene was completely lost from the mesoderm and ectoderm at the onset of gastrulation and became strongly expressed in the serosa just as it began to migrate out of the dorsal portion of the egg. It remained strongly expressed in the serosa cells as they encapsulated the embryo (not shown). This is interesting given the demonstrated role of the serosa in mediating innate immunity in other insect embryos [27], in combination with the ancestral role of NF-kB signaling in innate immunity.

Many of the genes expressed in the presumptive mesoderm showed a profound anterior to posterior progression in their expression pattern (e.g., *Nv-zinc-finger-homeodomain*, *Nv-zvh*, Fig. 4i–l, see also Fig. 4e, m). These genes appeared relatively late (in cycle 12) compared to *Nv-twi* and *Nv-sna*, and were only expressed in the mature, slug-shaped, presumptive mesoderm pattern. Initially, the anterior forked part of the pattern was visible, while expression was not seen in more posterior regions of the embryo (Fig. 4i). As the embryo aged, the expression domain of these genes extended towards the posterior (Fig. 4j, k), eventually filling out the presumptive mesoderm. By the time the expression domains reached their full posterior extent, gastrulation had begun in the anterior region where the expression of these genes was first detected (Fig. 4k, l). Since the movements of gastrulation follow the same AP progression, but slightly delayed in time, it is tempting to speculate that there is a functional correlation between these two events. This pattern also corresponds with the AP progression of numerous events in *Nasonia* embryonic development, including the syncytial mitotic waves [13], sex determination [28], and columnar gene activation [13].

In summary, not all mesoderm genes expand after BMP knockdown, and those that do appear to be downstream of the early responding genes (such as *Nv-twi* or *Nv-sna*). Many mesodermal genes show a distinct anterior to posterior progression of expression, indicating a significant interaction between the DV and AP patterning systems in *Nasonia*. Finally, it appears that only a small number of genes are expressed in the early ventral stripe that characterizes the conserved ventral genes *Nv-twi, Nv-sna, Nv-sim,* and *Nv-cact*.

### Some primarily ventral genes are also expressed in the serosa

Another (not mutually exclusive) set of ventrally expressed genes showed dorsal expression in addition to the strong ventral domain. Two of these genes, *Nv-cg2254* (not shown) and *Nv-inflated* (Fig. 4o) are involved in intercellular adhesion and morphogenesis, and may be crucial for facilitating the morphogenetic movements and/or tissue integrity of both mesoderm and serosa. Another is *Nv-heartless* (*Nv-htl*, Fig. 4n), an FGF signaling receptor, indicating that roles for this pathway in both extraembryonic and mesodermal tissue is an ancestral feature of the Holometabola, since the same is found in *Tribolium* [29].

### Ventrally expressed non-mesodermal genes

While most of the genes discussed above were expressed in stripes extending nearly from the anterior to the posterior pole, we found a set of four genes with highly restricted ventral domains. *Nv-crocodile* (*Nv-croc*) was expressed in both anterior and posterior ventral domains (Fig. 4p), along with (much weaker) stripes along the AP axis. By analogy to *Drosophila*, the ventral regions marked by *Nv-croc* might correspond to anterior and posterior midgut primordia, as do the corresponding domains in *Drosophila croc* [30, 31]. *Nv-meteorin* (Fig. 4q) was expressed in a circular domain overlapping the anterior *Nv-croc* domain, indicating it may be a specific marker of the anterior midgut primordium. Vertebrate *meteorin* homologs are involved in nervous system development [32] and thermoregulation [33], while it appears that this gene has been lost from the *Drosophila* lineage (JAL, personal observation).

At the posterior, three genes have restricted, but distinct, ventral domains (Fig. 4r–t). *Nv-neprilysin* encodes a putative metalloprotease, whose homologs have roles in vertebrates in activating ligands by cleaving them at specific residues, and are involved in numerous biological processes [34]. *Nv-tdrd7* encodes a protein containing a tudor domain and a LOTUS domain (very similar to the one found in *Nv-oskar* [35]). Its *Drosophila* homolog has been shown to repress transposon activity through Piwi-interacting RNA production in the germline [36]. After gastrulation, *Nv-tdrd7*-expressing cells are found in a region near where the gonads are thought to form (not shown), and in another experiment, we identified *Nv-tdrd7* as a transiently localized maternal germplasm component (Quan and Lynch, in preparation). It is not yet clear what the relationship (if any) between the ventral blastodermal expression and the apparent germline functions is. Finally, *Nv-CG14984* is the homolog of an uncharacterized *Drosophila* gene expressed in a broad, incomplete posterior ventral stripe (Fig. 4t).

### Genes repressed in the presumptive mesoderm

An additional expression class included two genes expressed late, and only at the outside edges of the presumptive mesoderm. One of them, *Nv-TollC* (Fig. 4u), is a paralog of the Toll gene required for ventral patterning, but it does show a DV patterning phenotype after pRNAi (JAL, personal observation). The other is a homolog of the *Drosophila* CG2254 (Fig. 4v), which encodes a putative oxidoreductase enzyme, and which is also expressed in the presumptive serosa. This is of particular interest, as the crucial roles of proteins regulating redox reactions in motile cell populations has been recently recognized [37].

Finally, three genes, *Nv-stardust* (not shown), *Nv-echinus* (Fig. 4x) and *Nv-multiple edematous wings* (*Nv-mew*) (Fig. 4y), are specifically expressed everywhere except the mesoderm, so they have been classified as mesodermally regulated genes. These regions of asymmetric concentration appear to prefigure regions of actin accumulation and tissue folding as gastrulation begins (JAL, personal observation). This indicates that these genes may play an important role in regulating the morphogenetic movements of the ectoderm and mesoderm at gastrulation.

### Laterally expressed genes

In both *Drosophila* and *Nasonia*, the lateral regions of the embryo give rise to the nervous system and to the larval ectoderm. Thirty-two of the genes we identified in our analysis were expressed in lateral domains, meaning that they are not expressed in the mesodermal or extraembryonic regions. There are several subcategories of lateral gene expression.

### New genes in the mesectoderm and ventral ectoderm

**Fig. 5:**
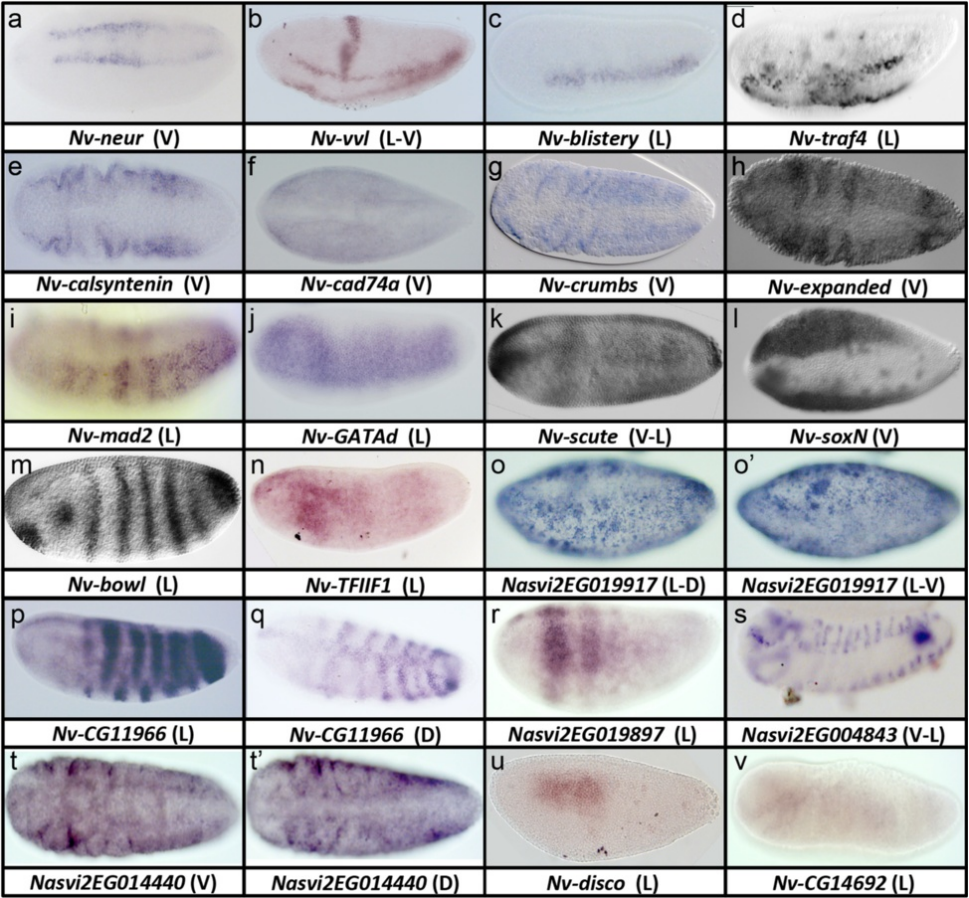
**a–v** Laterally expressed *Nasonia* genes. *D* dorsal view, *L* lateral view, *L-D* more lateral than dorsal view, *L-V* more lateral than ventral view, *V* ventral view *V-L* more ventral than lateral view. All embryos are in the last syncytial stage except **d**, **g**, **h**, and **t**, which are in the early stages of gastrulation. **o’** and **t’** are opposite focal planes of **o** and **t**, respectively

A set of seven genes was expressed in narrow ventrolateral domains. Most of these genes were strongly affected in *Nv-Toll* knockdown, and some showed upregulation in BMP knockdowns, indicating that they are regulated with similar logic to the ventral genes such as *Nv-twi* or *Nv-single minded* (*Nv-sim*). *Nv-neuralized* (*Nv-neur*) and *Nv-ventral-veins-lacking* (*Nv-vvl*) were expressed in mesectodermal patterns similar to *Nv-sim* (Fig. 5a, b). Stripes of *Nv-neur* and *Nv-vvl* flanking the mesoderm initially occupied domains with fuzzy borders spanning multiple cell widths. Over time these stripes resolved into a single cell width with no fuzziness, and were incorporated into the ventral midline at the end of gastrulation. *Nv-vvl* had an additional gap-gene-like expression at the anterior, which is likely regulated separately from the DV expression pattern. Both of these genes are conserved in *Drosophila*. However fly *vvl* is expressed embryonically only after gastrulation in the nervous system [38, 39], and fly *neur* is expressed in the mesoderm as well as in the mesectoderm [40].

Another set of genes was also expressed ventrolaterally, but with broader domains than the single cell of the ventral midline. This set included the known genes *Nv-vnd* and *Nv-rhomboid* (found only in annotation 1.2, not shown), as well as *Nv-blistery* (Fig. 5c) and *Nv-traf4* (Fig. 5d). *blistery* mediates epithelial adhesion and integrity [41], while *traf-4* is a signaling molecule involved in morphogenesis [42]. Interestingly, fly *blistery* is not regulated along the DV axis, while *Drosophila traf4* is expressed in the mesoderm rather than the ventral ectoderm.

### Broadly expressed lateral ectoderm genes

Twenty-two genes were expressed in broad lateral domains, taking up most of the space between the mesoderm and extraembryonic membranes. Seven of these were significantly reduced in BMP knockdown relative to water-injected embryos, which is consistent with our observation that BMP signaling plays the major patterning role outside of the mesodermal region. Eleven genes were detected only when the Toll and BMP datasets were compared. Nine of these were strongly reduced in the BMP case compared to Toll, while two increased in Toll relative to BMP knockdown. The former nine genes showed reduced (but not statistically significant) expression in BMP versus water-injected embryos, and increased (but insignificant) expression in Toll pRNAi versus water-injected embryos (Additional files 4 and 5).

Five of the 22 broad lateral genes are predicted to be involved in cell adhesion and morphogenesis. These are *Nasonia* homologs of *calsyntenin* (Fig. 5e), *cadherin 74a* (Fig. 5f), *crumbs* (Fig. 5g), *expanded* (Fig. 5h), and *wing blister* (not shown) [43–46]. None of the *Drosophila* homologs of these genes are expressed in similar patterns, indicating that these genes are good candidates for mediating the novel behavior of the ectoderm observed in higher hymenopterans.

A further eight broadly expressed genes encode transcription factors. These include homologs of *Mothers against dpp* (*Nv-mad2,* Fig. 5i), *GATA-D* (Fig. 5j), *achaete-scute* (Fig. 5k), *sox-neuro* (Fig. 5l), *bowel* (*Nv-bowl,* Fig. 5m), and the general transcription factor *TFIIF1* (Fig. 5n). The latter four genes have similar expression in *Drosophila* [47–50], indicating that they are a highly conserved set of ectodermal patterning genes, while *Nv-GATA-D* and *Nv-mad2* expression domains appear to be novel in *Nasonia*. Finally *Nv-CG11966* was expressed in a novel pair-rule type pattern where the lateral stripes were out of phase on the right and left sides of the embryo (Fig. 5p). This gene was also expressed in a dorsal, serosal stripe (Fig. 5q). Pair-rule and amnioserosal expression domains are also seen for the *Drosophila* homolog, but are comparatively weak.

The remaining nine of the broadly expressed genes include one gene with nucleotide metabolism function (*Nv-CG42249,* not shown), have unknown functions, or are novel genes. This includes four ankyrin domain-containing genes (discussed in detail below). *Nasvi2EG019917* has no clear similarity to any genes in other species, and possesses no obvious functional domains. It was expressed in a patchy pattern laterally and was repressed ventrally and dorsally (Fig. 5o-o'). Another novel gene, *Nasvi2EG019897*, was also expressed in a pair-rule pattern but was excluded from both the ventral and dorsal-most domains (Fig. 5r). *Nasvi2EG004843* was also repressed dorsally and ventrally, but was expressed in a single-segmental pattern (Fig. 5s). Finally, *Nasvi2EG014440* was expressed in a modulated pattern on the lateral side, which seemed to prefigure areas of folding of the epithelium (Fig. 5 t, t’).

### Dorsal ectoderm genes

There was also a set of four genes that was expressed dorsolaterally, including the known columnar gene *Nv-msh* [13], a homolog of the transcription factor *disconnected* (*Nv-disco,* Fig. 5u), a homolog of the potentially myosin-binding gene *CG14692* (Fig. 5v), and a novel ankyrin domain-containing gene (discussed later).

In summary, more than one third of the DV genes identified in this analysis were expressed in the lateral region of the embryo. This was despite the fact that we predicted that genes in this region would be the least affected by the pRNAis in terms of relative expression levels in the embryo. Several unexpected transcription factors, cell adhesion molecules, and cytoskeletal components were also detected. The former class contained good candidates for interacting with mesoderm to regulate the expansion of its domain, while the latter two classes contained good candidates for driving the novel morphometric movements of *Nasonia* gastrulation. Finally, expression patterns unlike any observed in *Drosophila* were found in the lateral domain of *Nasonia*, including a variation on a pair-rule pattern where the stripes on each side of the embryo were offset from each other.

### Dorsally expressed genes

The dorsal surface of the *Nasonia* embryo, much like that of *Drosophila*, gives rise to the extraembryonic membranes. Unlike the highly derived amnioserosa of *Drosophila*, *Nasonia* produces a full serosa that migrates and completely envelopes the embryo (it is not clear if an amnion is present in *Nasonia*). This embryonic region has the most genes expressed within it (33), possibly reflecting the complicated movements and later functions of the serosa.

Of the 33 genes expressed on the dorsal-most surface of the *Nasonia* embryo, 10 of them are transcription factors, 7 are components of signal transduction pathways, 3 are involved in cytoskeletal processes, 1 each are a protease or kinase, and the remaining 11 have unknown or novel functions. Almost all (27/33) were significantly reduced in BMP knockdown versus water-injected embryos, which is expected given that this region is the main patterning domain of the BMP pathway. The remaining genes were detected in the Toll versus BMP knockdown comparison.

**Fig. 6:**
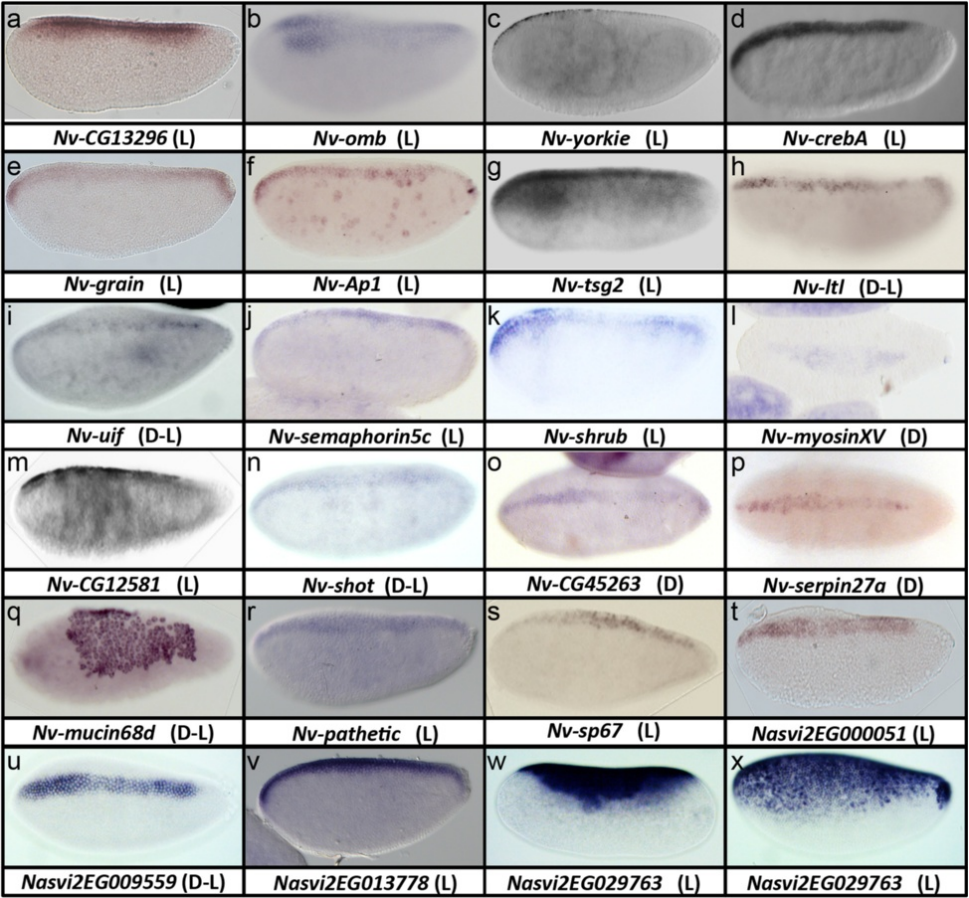
**a–x** Dorsally expressed genes. *D* dorsal view, *D-L* more dorsal than lateral view, *L* lateral view, *L-D* more lateral than dorsal view. All embryos are in the last syncytial blastoderm stage, except **q**, which has just begun gastrulation, and **w**, which is in the pre-blastoderm stage

Among the transcription factors, orthologs of the well-known *Drosophila* amnioserosa genes *zerknullt*, *tailup*, and *dorsocross* are significantly downregulated in BMP pRNAi (Additional file 4), and are expressed in narrow stripes along the dorsal midline of the embryo [13]. *Nasonia* possesses numerous other factors that show this pattern that are not expressed specifically in the fly embryo. These include a PR-zinc finger protein related to *CG13296* (Fig. 6a), which was the most strongly affected gene in this analysis, being reduced more than 18-fold after BMP knockdown. Close behind in magnitude of knockdown (14×) was *Nasonia optomotor-blind* (*Nv-omb*), a T-box transcription factor that was initially expressed in a broad dorsal stripe that then split, forming two stripes that flanked the serosal domain (Fig. 6b). It is interesting to note that *omb* is a direct target of BMP signaling in many contexts in *Drosophila*, for example, the imaginal discs [51], but not in the embryo. Other transcription factors included *Nasonia* homologs of *yorkie* (Fig. 6c, this transcript was apically localized within serosal cells), *creb-A* (Fig. 6d), *grain* (Fig. 6e), and *AP-1* (Fig. 6f).

Among the eight signaling components expressed on the dorsal half of the embryo, four of them are predicted to be involved in BMP signaling. This strongly suggests that BMP signaling is highly self-regulatory in *Nasonia*, and could help explain how it performs its patterning role. Aside from the previously described *Nv-punt* (receptor) and *Nv-cv2* (membrane-bound modulator, [52]), one *crossveinless/tsg* paralog (*Nv-tsg2,* Fig. 6g) and an ortholog of *larval translucida* (*Nv-ltl*, a secreted BMP modulator [53], Fig. 6h) were expressed in dorsal domains. *Nv-tsg2* is particularly interesting, as it was broadly and dynamically expressed, and might serve as a sensitive transcriptional readout of BMP activity. The other four signaling genes were *Nv-uninflatable* (*Nv-uif,* Notch, Fig. 6i), *Nv-semaphorin* (semaphorin, Fig. 6j), *Nv-yorkie* (hippo, Fig. 6c), and *Nv-shrub* (ESCRT, Fig. 6k).

The *Nasonia* serosa undergoes dramatic morphogenetic movements. It erupts out of the dorsal epithelium, and rapidly expands by crawling over the surface of the ectoderm until the embryo is completely encapsulated (Fig. 1, [13]). We have identified four genes expressed exclusively on the dorsal side that potentially are employed to bring about these movements. They are *Nasonia* homologs of *Myosin XV* (*Nv-myosinXV,* Fig. 6l), the pleckstrin homology domain-containing gene *CG12581* (Fig. 6m), the actin-microtubule cross-linking gene *short stop* (*Nv-shot,* Fig. 6n), and the immunoglobulin domain-containing *CG45263* (Fig. 6o). As mentioned above, several genes with mesodermal expression also showed expression in the serosal anlage. These may be genes that are general to motile cells.

There were five functional classes represented by single genes on the dorsal side of the embryo: a transaminase enzyme (*Nv-CG8745,* not shown), a serine protease inhibitor (*Nv-serpin27a*, Fig. 6p), a mucin (*Nv-mucin68d*, Fig. 6q) and an amino acid transporter (*Nv-pathetic*, Fig. 6r), and a serine protease (*Nv-sp67*, Fig. 6s). Finally, there were seven dorsally expressed genes that lack clear orthologs in *Drosophila*. Two of them were ankyrin domain-containing genes, as discussed below. Four were expressed in typical narrow stripes (Fig. 6s–v), indicative of potential novel roles in the specification and/or function of the serosa [*Nv-sp67* (Fig. 6s), *Nasvi2EG000051* (Fig. 6t), *Nasvi2EG009559* (Fig. 6u), and *Nasvi2EG013778* (Fig. 6v).] Finally, one gene was expressed broadly and strongly on the dorsal side from very early stages (possibly maternally) and through gastrulation (*Nasvi2EG029763,* Fig. 6w, x).

### Gap-like patterns at the poles

**Fig. 7:**
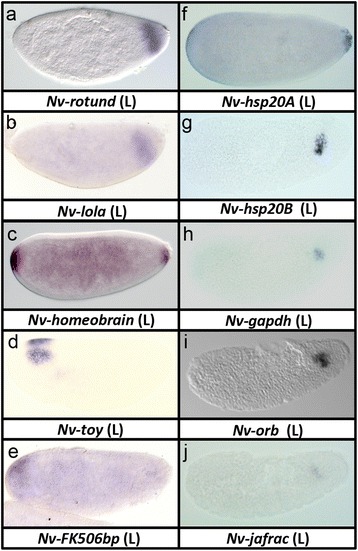
Genes with anteroposterior gap-like domains and germline expression. Anteroposterior (**a**–**d**) and germline (**e**–**j**) expression patterns were detected unexpectedly in our analysis. All are shown in lateral (*L*) view. **a**–**f** are in the final syncytial blastoderm stage, while **g**–**j** have completed gastrulation

Our experimental approach was designed to identify genes differentially expressed along the DV axis. However, it is clear that the two orthogonal axes are not completely independent, and in fact there is much cross-talk between the two axial patterning pathways, particularly at the poles. Accordingly we have identified a set of terminally expressed genes, including the previously known genes *Nv-tailless*, *Nv-orthodenticle1*, and *Nv-orthodenticle2*. Other genes expressed at the extreme poles include *Nv-rotund* (Fig. 7a), *longitudinals lacking* (*Nv-lola, *Fig. 7b), *Nv-homeobrain* (Fig. 7c), *Nv-twin-of-eyeless* (*Nv-toy*, Fig. 7d), and FK506-binding protein (*Nv-FK506bp,* Fig. 7e). Of these, only *toy* is expressed in a comparable domain in *Drosophila* [54].

### Early zygotic germline gene expression

We also uncovered an unexpected set of genes that is zygotically expressed in the primordial germ cells. Two *heat shock protein 60* paralogs (*Nv-hsp60A, Nv-hsp60B,* Fig. 7f, g), a *glyceraldehyde-3-phosphate dehydrogenase* homolog (*Nv-gapdh*, Fig. 7h), the *Nasonia oo18 RNA-binding protein* (*Nv-orb,* Fig. 7i), and a homolog of the thioredoxin *jafrac* (*Nv-jafrac,* Fig. 7j) all showed statistically significant reduction after Toll knockdown. None of these genes showed expression in the oosome (germ plasm), indicating that they are activated zygotically, and are at least partially reliant on Toll signaling (directly or indirectly) for their activation. The reason for this is not clear, as no clear effect on the expression levels of these genes was detectable when comparing wt and Toll knockdown embryos. However, it does point to the possibility that other zygotic germline genes may be in the set of marginal genes that have not yet been analyzed. Interestingly, the *Drosophila* homologs of all of the germline genes discovered here are also expressed zygotically in the fly germline [50, 55, 56].

### Genes lost in the *Drosophila* lineage

While the vast majority (88/110) of the genes we have described here have clear *Drosophila* homologs, the remainder are novel relative to *Drosophila*. One of these, *Nv-meteorin* (Fig. 4q), is found in vertebrates and most other insects, including mosquitos, indicating a recent loss from the fly genome. Another, *Nv-myosinXV* (Fig. 6l) has clear orthologs in vertebrates and hemimetabolous insects, but this gene appears to have been lost in the non-hymenopteran lineage after the split from the rest of the Holometabola (JAL, personal observation). An additional gene (*Nasvi2EG013778*, Fig. 6v) is found in many insect lineages, but is not found in *Drosophila*, or in other metazoan lineages outside of insects. Understanding the functions of these genes in *Nasonia* and other insects could give insights into the evolution of *Drosophila* by indicating which molecular functions were made unnecessary or redundant in the course of the evolution of its lineage.

### Hymenoptera-specific genes

Four of the genes found in *Nasonia* are found in distantly related Hymenoptera such as bees and ants, but nowhere else in the tree of life [*Nasvi2EG010608* (not shown), *Nasvi2EG000051* (Fig. 6t), *Nasvi2EG009559* (Fig. 6u), *Nasvi2EG004843* (Fig. 5s)]. We describe these as Hymenoptera-specific genes. We found four potentially novel genes unique to *Nasonia*, or at least not found in ants or bees or anywhere else more distant in the tree of life [*Nasvi2EG014440* (Fig. 5t)*, Nasvi2EG029763* (Fig. 6w, x)*, Nasvi2EG019917* (Fig. 5o), and *Nasvi2EG019528* (not shown)]. We cannot exclude that homologs of these genes are found in other parasitic wasps.

### Wasp-specific ankyrin domain-containing genes

**Fig. 8:**
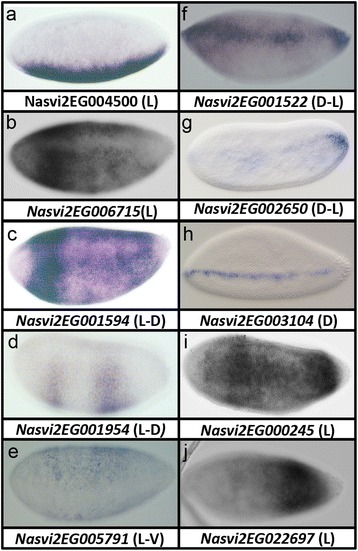
**a-j** Ankyrin domain-containing genes with distinct dorsoventral expression patterns. All are in the last syncytial stage prior to gastrulation. *D* dorsal view, *D-L* more dorsal than lateral view, *L* lateral view, *L-D* more lateral than dorsal view

A particularly fascinating set of novel genes encodes proteins that possess a characteristic set of ankyrin repeats (Fig. 8). When these are used as BLAST queries, the top hits are typically a combination of related *Nasonia* genes and genes found outside the Metazoa, including bacteria and fungi (JAL, personal observation). Thus, it appears that the *Nasonia* ankyrin domain-containing genes we have found are closely related to each other, and are quite distinct from genes found in other insects (including fellow hymenopterans such as ants and bees).

Genes of this class were one of the great surprises found in the sequencing of the *Nasonia* genome. It has previously been determined that the *Nasonia* genome contains the largest complement of ankyrin domain-containing genes so far annotated [57]. Particularly interesting were ankyrin domain-containing genes that also posses the so-called PRANC (Pox proteins Repeats of ANkyrin, C-terminal) domain. PRANC-containing genes appear to have originated in poxviruses, where one of the functions of the PRANC domain is to manipulate the NF-kB function of eukaryotic hosts [58]. This association with NF-kB is tantalizing, as Dorsal is a transcription factor of this class. Whether this function is retained and relevant in *Nasonia* remains to be tested.

The PRANC domain-containing genes appear to have entered the *Nasonia* genome through a *Wolbachia* endosymbiont intermediate, as the *Nasonia* genes are highly related to those still found in their endosymbionts [57]. While many of the wasp-specific ankyrin genes lack identifiable PRANC domains, they are highly similar in the rest of their sequence, indicating that they had a relatively recent common origin. A similar pattern of apparent loss of the PRANC domains is also observed in poxviruses themselves [59]. It is not clear how many horizontal transfers are responsible for the current array of more than 250 ankyrin domain-containing genes, but clearly gene duplication played a major role in amplifying this gene family.

We found a single ankyrin domain-containing gene, *Nasvi2EG004500*, expressed ventrally in a broad, dynamic domain that was similar to genes such as *Nv-twi* or *Nv-sna*. However, expression of this gene remained pole to pole, instead of taking on the typical slug-shape of the presumptive mesoderm (Fig. 8a). Two genes (*Nasvi2EG006715* and *Nasvi2EG001594* and) were broadly expressed laterally, with a more intensely staining “gap-like” domain near the head primordium (Fig. 8b, c). One ankyrin domain protein was expressed in two broad stripes that were repressed on the dorsal side (*Nasvi2EG001954*, Fig. 8d), while another was broadly expressed but repressed in a wide ventral domain (*Nasvi2EG005791*, Fig. 8e). Three genes were expressed on the dorsal side of the embryo. One (*Nasvi2EG001522*, Fig. 8f) was expressed broadly at the poles, and in a narrow stripe in the main trunk of the embryo. Another (*Nasvi2EG002650*, Fig. 8g) was highly expressed at the extreme poles (anterior domain out of focus) of the embryo, and in an incomplete stripe along the dorsal midline. Most astoundingly, *Nasvi2EG003104* was expressed in a narrow, single nucleus-wide domain (Fig. 8h). Finally, two ankyrin domain-containing genes were expressed in broad posterior domains: *Nasvi2EG000245* (Fig. 8i) and *Nasvi2EG022697* (Fig. 8j). It is interesting to note that almost all of the ankyrin domain-containing gene expression domains are unique, indicating that they have all independently evolved their own novel *cis*-regulatory elements driving novel patterns in the embryo.

Three of the 10 ankyrin genes with DV expression had unambiguous PRANC domains at their C-termini (*Nasvi2EG001522*, *Nasvi2EG003104*, and *Nasvi2EG002650*), while the rest appeared to lack them. Given the similarity of these 10 genes, we propose that in the process of gene family amplification, some of the paralogs may have lost the PRANC domain, while maintaining a core characteristic ankyrin repeat domain. Whatever the evolutionary history of these genes, it is clear that they are novel components of the *Nasonia* genome, yet they seem to have been stably incorporated into the *Nasonia* DV patterning GRN at different positions along the axis.

Other cases of horizontal gene transfer have been detected in insects [60], some of which are functional in providing an insect with novel biochemical capabilities that aid in defense [61]. In *Drosophila ananassae*, a whole *Wolbachia* chromosome has been transferred to the nuclear genome, but appears to have little expression and no apparent function [62]. In contrast, the *Nasonia* genes are expressed robustly and represent, to our knowledge,  the first example of horizontally transferred genes being incorporated into a patterning network.

### Genes with an unexpected response to pRNAi

**Fig. 9:**
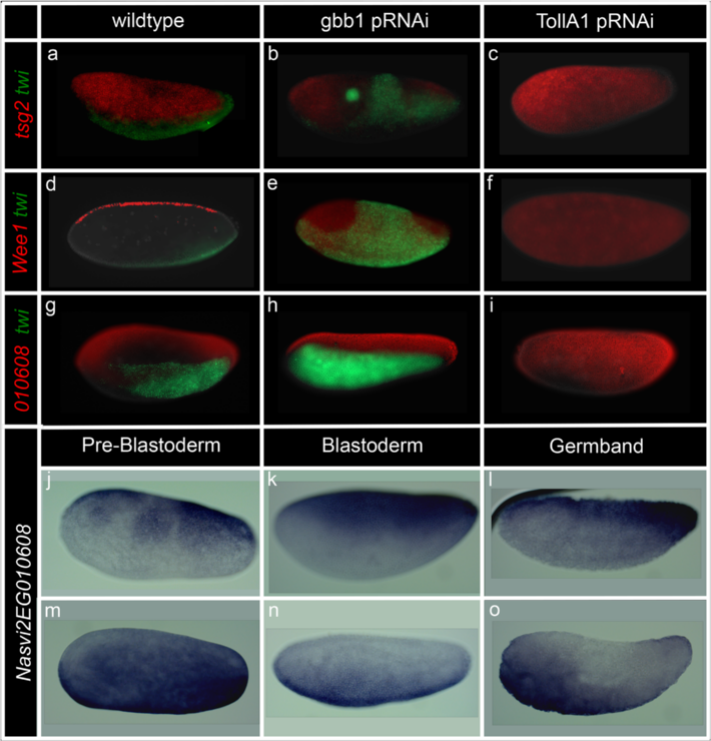
Characterization of *Nasonia* transcripts with unexpected responses to pRNAi. **a**–**i** Simultaneous detection of transcript of interest (*red*), *twist* (*green*) (to confirm the knockdown: expansion of *twist* in gbb1 KD and loss of *twist* in TollA1 KD), and DAPI (*white*) in blastoderm stage embryos. Changes in expression of *tsg2* (**a**–**c**), *Wee1* (**d**–**f**), and *Nasvi2EG010608* (**g**–**i**) between wild type (**a**, **d**, **f**), BMP knockdown (**b**, **e**, **g**), and Toll knockdown (**c**, **f**, **i**) embryos are observed. **j**–**o** non-fluorescent detection of *Nasvi2EG029763* transcripts. Dorsal (**j**–**l**) and ventral (**m**–**o**) expression is observed prior to the formation of the syncytium (**j**, **m**), through all blastoderm stages (**k**, **n**), and persists through gastrulation and germband elongation (**l**, **o**). In all frames, anterior is left and dorsal is up

The vast majority of the genes we have examined in this project follow the expected correlation between control expression patterns with a change in levels after pRNAi (i.e., ventrally expressed genes are strongly reduced in Toll knockdown, and dorsally expressed genes are strongly reduced in BMP knockdown). However, there were a few genes whose normal expression patterns were not predicted by their behavior in the RNAi analyses. Two of these genes, the *Nasonia* homologs of a modulator of BMP protein signaling, *twisted gastrulation* (*Nv*-*tsg2*), and a G2 checkpoint kinase, *wee1* (*Nv-wee1*), exhibited dorsal expression patterns (Fig. 9a, d). A reduction in transcript levels after BMP knockdown (by ~50 % and 20 % respectively, Additional file 4) is consistent with the observed dorsal expression pattern; however, increases in transcript levels after Toll knockdown were also observed (by ~50 % and 60 %, Additional file 4). This result was unexpected, as up until this point, all dorsally expressed genes had been observed to act independently of Toll regulation. To better elucidate the regulation of these two genes, in situ hybridization experiments of these transcripts were performed on BMP- and Toll knockdown embryos. In wt embryos, *Nv-tsg2* was expressed in a broad dorsal domain (Fig. 9a). In BMP knockdown embryos, *tsg2* expression at the anterior pole expanded ventrally, while trunk expression was greatly reduced (Fig. 9b). In Toll knockdown embryos, expression of *tsg2* was nearly ubiquitous, expanding ventrally almost to the ventral midline (Fig. 9c). Both of these results are consistent with the quantified expression levels observed (increased expression in Toll knockdown, and decreased expression in BMP knockdown), and suggest regulation from both the Toll and BMP pathways.

In wild-type embryos, *Nv-wee1* was expressed in a narrow stripe along the dorsal midline (Fig. 9d); however, in both BMP and Toll knockdown embryos, *Nv-wee1* was expressed ubiquitously throughout the embryo (perhaps reduced or absent at the ventral midline in Toll) (Fig. 9e, f). This in situ data agrees with the observed relative change in expression level in Toll knockdown embryos, but contradicts observations in BMP knockdown embryos. Unlike with *tsg2*, *Wee1* does not appear to be inhibited by *twist*, and in fact expands ventrally as *twist* expands dorsally. The loss of localization seen in the absence of both BMP and Toll suggest that neither are required for activation, but perhaps function to indirectly restrict its domain normally through downstream effectors.

Another gene whose normal expression pattern was not predicted by its behavior in the RNAi analyses was a novel gene, *Nasvi2EG010608*. Expression decreased markedly after Toll knockdown (by ~80 %, Additional file 4) and only slightly decreased after BMP knockdown (by ~10 %, Additional file 4), leading to the prediction that this novel gene should have an expression pattern localized to the ventral half of the embryo. However, only half of the wild-type embryos examined showed a ventral pattern, while the other half showed a dorsally localized pattern (Fig. 9g, j–o). This ratio was maintained in every stage from the first few nuclear divisions up through germband elongation (Fig. 9j-o). No correlation to sex was observed, since eggs from virgin females (all males) and from fertilized females (normally a 9:1 female to male ratio) showed the same 50/50 ratios of dorsal and ventrally expressed *Nasvi2EG010608* (data not shown).

Again, in situ hybridization experiments of these transcripts were performed in the two knockdown conditions. In BMP knockdown embryos, expression was reduced to a slightly smaller ventral (not shown) or dorsal (Fig. 9h) domain. In Toll knockdown embryos, expression expanded dramatically to near ubiquity, with weak or no expression along the ventral midline (Fig. 9i). While in situ data from the pRNAi embryos confirmed the slight reduction seen in the quantification of BMP knockdown expression levels compared to control, it seemed to contradict the reduced expression observed from the Toll RNAi-RNAseq analyses. Additionally, the in situ data failed to shed light on the variation of expression observed in wild-type embryos. Further experiments will be done to determine the cause of this dynamic expression pattern.

### Limitations of the analysis

Nearly all of the genes that were known to be expressed in patterns along the DV axis of the *Nasonia* embryo were found with high confidence in our analysis. However, a few were overlooked in one or both of the DE analyses based on the two transcriptome annotations we used. The lateral portions of the embryo were always the clear weak point of our approach, as genes expressed in these regions were predicted to require low amounts of either Toll or BMP input, so would be least sensitive to reduction of these pathways. Thus, we were pleasantly surprised to be able to identify a large number of genes with specific lateral expression patterns. However, most of these were initially classified as marginal, and were largely at the edge of our statistical significance and fold change cutoffs. We would not be surprised to find that there may be additional laterally expressed genes, especially given that several genes known to have lateral patterns were not identified in our analyses.

Particularly striking was that *Nv-brk* was not found in either analysis. This gene is highly expressed laterally, and is clearly regulated by both Toll and BMP [14]. However, the RNAseq approach can only detect changes in global levels of RNA expression, and not redistribution. Another known gene that did not appear in our analysis is *Nv-ind*, which is the most “lateral” member of the columnar gene group [13] , and is expressed in a region furthest away from the peaks of activity of the two signaling pathways patterning the *Nasonia* DV axis, and thus may be insensitive to changes to one gradient or the other.

Another set of genes that might have been missed by our analysis is composed of genes that have multiple domains of expression, which reduces the overall magnitude of DE after single knockdowns. For example, the ventral domain of *Nv-snail* is strongly affected by *Nv-Toll* knockdown, but the magnitude of the expression difference between water and *Nv-Toll* expression were much less than for comparable genes like *Nv-twi*. This is likely due to high expression of *Nv-sna* in the yolk nuclei [13], which is not affected by *Nv-Toll* pRNAi. Similarly, genes expressed both ventrally and dorsally show reduced magnitudes of DE in the RNAi cases, and we propose that this explains why genes like *Nv-tgfα* were missed. These genes have late ventrolateral expression similar to (but temporally delayed relative to) *Nv-vnd*, and dorsal stripes flanking the serosal domain (JAL, personal observation). Toll pRNAi has a relatively weak input into genes expressed like *Nv-vnd* (compared to those in the mesoderm), so the magnitude of change is likely to less than 100 % on the ventral side. This in combination with a significant amount of remaining expression on the dorsal side could lead to an overall reduction below our ~1.5× cutoff. In BMP knockdown, the same factors are in play with the additional caveat that the ventral domain of this type of gene could increase, offsetting the reduction of mRNA produced on the dorsal side.

Finally, errors and omissions in the genome sequence, assembly, or annotation can lead to some genes being missed. Despite all of this, given the large number of genes that were detected, we believe the vast majority of the participants in the *Nasonia* DV GRN have been identified.

### Comparison to *Drosophila*

A similar experiment was conducted in *Drosophila*, using microarray technology to detect DE of transcripts along the entire DV axis [7, 25]. Many novel insights were gained in that study (and subsequent ones taking different technological or methodological approaches [63–66]), including the discovery of new FGF pathway ligands. In addition, comprehensive analyses of embryonic gene expression identified further genes expressed in different patterns along the axis [50, 67]. The evolutionary significance of the *Drosophila* work was until recently not entirely clear, as it represented a single comprehensive data point. With our data, and the basal position of *Nasonia* within the Holometabola, we can now begin to propose a conserved core set of tissue specification genes for mesoderm, ectoderm, and extraembryonic tissues, as well as generate hypotheses about novel gains and losses of genes from the DV patterning GRN within the Holometabola.

The transcription factors Twist, Snail, Six4, and Zinc Finger Homeodomain; the FGF signaling components Stumps and Breathless; the axon guidance molecule Netrin; and the integrin Inflated are conserved mesoderm factors between *Nasonia* and *Drosophila*. Eight transcription factors (Vnd, Sim, Sox-N, Bowl, TFIIF1, Msh, Brk, Ind) and another integrin (Multiple Edematous Wings, *mew*) have conserved ectodermal expression patterns. In the extraembryonic and dorsal embryonic region, 11 genes have conserved expression. This includes six transcription factors (Tail-up, Grain, Pannier, Dorsocross, Zen, and Araucan), two BMP modulators (Crossveinless-2 and Twisted Gastrulation), the immunoglobulin domain protein CG43462*,* the membrane-bound signaling molecule Semaphorin5c, and the enzyme RACE. In total, 18 of the 28 conserved genes are transcription factors. This is consistent with a model in which specific transcription factors have conserved roles in specifying tissue fates that are broadly conserved in evolution. On the other hand, molecules involved in cell structure and behavior are less evolutionary stable, as the behaviors of, and interactions among, embryonic tissues change much more frequently than the presence of the tissue itself.

To better understand how our *Nasonia* results compare to what is known in *Drosophila*, we compiled a list of 278 genes with distinct expression along the DV axis of the fly embryo at the late blastoderm stage, combining the microarray, chromatin immunoprecipitation sequencing, and high-throughput in situ data available in *Drosophila* (see Additional file 6 and Methods for how this list was generated). This list is likely to be more comprehensive than the set of confirmed DV expressed genes we have found in *Nasonia* and likely includes expression patterns and levels that we excluded from our analysis. For example, some genes with significant DE after RNAi were excluded from our analysis due to very low FPKM values in wild type. A handful of *Drosophila* homologs of such genes have DV expression, indicating that this class of lowly expressed genes may be a fruitful source of DV patterning genes in future analyses in *Nasonia*. Overall, this analysis showed again that there is a large divergence in DV expressed genes between wasp and fly, as only 35/278 genes with patterned DV expression in fly have homologs that show DE in at least one comparison in our *Nasonia* data.

Sixty-six (23 %) of the *Drosophila* DV genes have no clear *Nasonia* homologs, which is similar to the 18 % of *Nasonia* genes without clear fly orthologs (Additional file 5).Twenty-four of the 66 (36 %) are only found in diptera, and 17 of these 24 are found only within the *Drosophila* lineage and its close fly relatives. This is in strong contrast to what we found in *Nasonia,* where 17 of the 23 genes without fly orthologs are unique to the Hymenoptera, and 14 of these 17 are so far only found in *Nasonia*. It remains to be seen if these differences in lineage-specific novelty reflect a true difference in evolutionary pattern, or if it is an artifact of different levels of sampling between the two lineages.

Five of the genes without *Nasonia* orthologs are found in Coleoptera and/or Lepidoptera, indicating that these genes originated after the split between Hymenoptera and the rest of the holometabolan lineage. The remaining 37 of the 66 *Drosophila* DV genes without *Nasonia* homologs (13 % overall) provisionally represent wasp lineage gene losses. This is more than two-fold higher than the ~5 % of *Nasonia* DV genes whose lack of fly homologs is due to loss along the fly lineage.

### Comparison to the beetle *Tribolium castaneum*

A conceptually similar experiment was recently published for the beetle *Tribolium castaneum* [68]. Given the quite different blastoderm fate map in *Tribolium*, and the fact that much DV patterning takes place well after gastrulation [16, 69, 70], direct comparisons with *Nasonia* and *Drosophila* are not straightforward. Despite this difficulty, the *Tribolium* experiments identified several new conserved insect DV components: the transcription factor ZFHD, the integrin Inflated, the FGF signaling component Dof, and the Notch pathway component Uif. Further in-depth comparisons among wasp, beetle, and fly will begin to give a clearer understanding of how embryonic patterning networks have evolved among the holometabolous insects.

## Conclusions

We have shown here that, apart from a conserved core of mostly transcription factors, the GRNs for DV patterning and early morphogenesis have diverged significantly between the wasp *Nasonia* and the fly *Drosophila*. This fits well with the observations that both the upstream patterning networks, and the downstream morphogenetic events of gastrulation are quite divergent. At present we cannot tell which network is more representative of the ancestral state of the Holometabola, or indeed whether they are both highly divergent. With the advent of increasingly cost-effective large-scale sequencing technologies, and broadly applicable functional approaches, many more taxa can be sampled and characterized at a deep level. This should allow hypothesis of the pattern and direction of phylogenetic change to be tested robustly. Our observation that many lineage restricted genes have been incorporated into the *Nasonia* DV GRN raises the question of whether such novelty is a common feature of developmental patterning systems, and what role, if any, natural selection plays in setting up the early body plan. Again, broad and deep sampling of taxa at varying phylogenetic distances can shed light on this question and should be a high priority research area where insects can make a uniquely powerful contribution.

## Additional files

Additional file 1: Methods: Compilation of scripts used in TopHat and Cufflinks analyses. (DOCX 98 kb) Additional file 2: Gene expression cuffdiff output (gene_exp.diff) using *Nasonia* transcriptome annotation 2.0. This result was used as the main basis for choosing genes to further validate with in situ hybridization. See sections I–IV of the Additional file 1: Methods for how this result was produced. (XLSX 6260 kb) Additional file 3: Gene expression cuffdiff output (gene_exp.diff) using *Nasonia* transcriptome annotation 1.2. This supplemented the main result given in Additional file 2, due to some discrepancies between the two annotations. See sections V–VI for how this result was produced. (XLSX 138 kb) Additional file 4: Annotation of all genes where significant differential expression (DE) was detected in at least one comparison. Each gene has two rows: one for the Toll vs. wt comparison, and one for the BMP(dpp) vs. wt. All data is from the annotation 2.0 file regardless of whether DE was detected only in the annotation 1.2 analysis. Columns B–H are essential information from the gene_exp.diff file. Column I = pattern of DE of the gene; Column J = primary embryonic expression pattern; Column K = any additional patterns of expression; Column L = general molecule type; Column M = whether gene has clear orthologs in flies, insects, other animals, or is novel; Column N = association with a signaling pathway; Column O = cell biological function of gene; Column P = fly ortholog (if applicable); Column Q = whether gene is expressed in a similar pattern in *Drosophila*. (XLSX 158 kb) Additional file 5: Annotation of genes with confirmed expression patterns. This file is a subset of Additional file 4, and contains only genes for which patterned expression was detected. Classifications in this file were used to produce the charts in Fig. 3. (XLSX 66 kb) Additional file 6: Dorsoventrally regulated genes in *Drosophila melanogaster*. Column A: CG number corresponding to each gene. When available, CG is also a hyperlink to the corresponding BDGP page where in situ images can be found. Column B: Gene name. Column C: Gene symbol. Column D: Primary localization of RNA transcript at stage 4–6, determined from in situ data. Column E: Molecular function category determined from experimental data collected by flybase.org. Column F: Detection of *Nasonia* ortholog in RNAi-RNAseq differential expression data [No = not significant, − = no ortholog to detect, toll vs. dpp = significant differential expression between dorsalized and ventralized embryos, wt vs. dpp = significant differential expression between dorsalized and wt embryos, wt vs. toll = significant differential expression between ventralized and wt embryos]. Column G: Corresponding *Nasonia vitripennis* orthologs as reported by flybase.org [N/A = no known ortholog]. Column H: Novelty status of *Drosophila* genes for genes without *Nasonia* orthologs. (XLSX 43 kb)
