# Supplementary material for: Global analysis of dorsoventral patterning in the wasp Nasonia reveals extensive incorporation of novelty in a regulatory network
Source: BMC Biol. 2016 Aug 1;14:63. doi: 10.1186/s12915-016-0285-y (PMC4968023; doi:10.1186/s12915-016-0285-y)
Supplement: Additional file 1: — Methods: Compilation of scripts used in TopHat and Cufflinks analyses. (DOCX 98 kb) [file 12915_2016_285_MOESM1_ESM.docx]

**I. Tophat2 commands annotation 2.0:**

277gbb1611th20.ph

#!/bin/bash -l

#PBS -q default

#PBS -l nodes=1:ppn=16

#PBS -l mem=64gb

#PBS -l walltime=8:00:00

#PBS -j oe

#PBS -A AG-Roth

export PATH=$HOME/bowtie2-2.0.0-beta6:$HOME/tophat-2.0.3.Linux_x86_64:$HOME/newtophat/samtools-0.1.18:$PATH

echo$PATH

#export NCORES=`cat $PBS_NODEFILE | wc -l`

#export OMP_NUM_THREADS=$NCORES

OUTDIR=/scratch/jlynch/zippednvngs

cd $OUTDIR

tophat2 -p 16 -r 0 -I 10000 -o 277gbb1611th20 -G /scratch/jlynch/zippednvngs/nv2clean.gff --no-novel-juncs ass1nvbowtie2 /scratch/jlynch/zippednvngs2/SN7640117_5883_Gbb11611_1_sequence.fq.gz /scratch/jlynch/zippednvngs2/SN7640117_5883_Gbb11611_2_sequence.fq.gz

277dpp2610th20.ph

#!/bin/bash -l

#PBS -q default

#PBS -l nodes=1:ppn=16

#PBS -l mem=64gb

#PBS -l walltime=4:00:00

#PBS -j oe

#PBS -A AG-Roth

export PATH=$HOME/bowtie2-2.0.0-beta6:$HOME/tophat-2.0.3.Linux_x86_64:$HOME/newtophat/samtools-0.1.18:$PATH

echo$PATH

#export NCORES=`cat $PBS_NODEFILE | wc -l`

#export OMP_NUM_THREADS=$NCORES

OUTDIR=/scratch/jlynch/zippednvngs

cd $OUTDIR

tophat2 -p 16 -r 0 -I 10000 -o 277dpp2610th20 -G /scratch/jlynch/zippednvngs/nv2clean.gff --no-novel-juncs ass1nvbowtie2 /scratch/jlynch/zippednvngs2/SN7640117_5880_dpp2610_1_sequence.fq.gz /scratch/jlynch/zippednvngs2/SN7640117_5880_dpp2610_2_sequence.fq.gz

197h2o805tophat.ph

#!/bin/bash -l

#PBS -q default

#PBS -l nodes=1:ppn=16

#PBS -l mem=64gb

#PBS -l walltime=4:00:00

#PBS -j oe

#PBS -A AG-Roth

export PATH=$HOME/bowtie2-2.0.0-beta6:$HOME/tophat-2.0.3.Linux_x86_64:$HOME/newtophat/samtools-0.1.18:$PATH

echo$PATH

#export NCORES=`cat $PBS_NODEFILE | wc -l`

#export OMP_NUM_THREADS=$NCORES

OUTDIR=/scratch/jlynch/zippednvngs

cd $OUTDIR

tophat2 -p 16 -r 0 -I 10000 -o 197h2o805 -G /scratch/jlynch/zippednvngs/nv2clean.gff --no-novel-juncs ass1nvbowtie2 /scratch/jlynch/zippednvngs2/SN7640117_5881_H2O0805_1_sequence.fq.gz /scratch/jlynch/zippednvngs2/SN7640117_5881_H2O0805_2_sequence.fq.gz

197h2o1704tophat.ph

#!/bin/bash -l

#PBS -q default

#PBS -l nodes=1:ppn=16

#PBS -l mem=64gb

#PBS -l walltime=4:00:00

#PBS -j oe

#PBS -A AG-Roth

export PATH=$HOME/bowtie2-2.0.0-beta6:$HOME/tophat-2.0.3.Linux_x86_64:$HOME/newtophat/samtools-0.1.18:$PATH

echo$PATH

#export NCORES=`cat $PBS_NODEFILE | wc -l`

#export OMP_NUM_THREADS=$NCORES

OUTDIR=/scratch/jlynch/zippednvngs

cd $OUTDIR

tophat2 -p 16 -r 0 -I 10000 -o 197h2o1704 -G /scratch/jlynch/zippednvngs/nv2clean.gff --no-novel-juncs ass1nvbowtie2 /scratch/jlynch/zippednvngs2/SN7640117_5878_H2O1704_1_sequence.fq.gz /scratch/jlynch/zippednvngs2/SN7640117_5878_H2O1704_2_sequence.fq.gz

277TollA1611th20.ph

#!/bin/bash -l

#PBS -q default

#PBS -l nodes=1:ppn=16

#PBS -l mem=64gb

#PBS -l walltime=4:00:00

#PBS -j oe

#PBS -A AG-Roth

export PATH=$HOME/bowtie2-2.0.0-beta6:$HOME/tophat-2.0.3.Linux_x86_64:$HOME/newtophat/samtools-0.1.18:$PATH

echo$PATH

#export NCORES=`cat $PBS_NODEFILE | wc -l`

#export OMP_NUM_THREADS=$NCORES

OUTDIR=/scratch/jlynch/zippednvngs

cd $OUTDIR

tophat2 -p 16 -r 0 -I 10000 -o 277TollA1611th20 -G /scratch/jlynch/zippednvngs/nv2clean.gff --no-novel-juncs ass1nvbowtie2 /scratch/jlynch/zippednvngs2/SN7640117_5882_TollA-1611_1_sequence.fq.gz /scratch/jlynch/zippednvngs2/SN7640117_5882_TollA-1611_2_sequence.fq.gz

277TollA2610th20.ph

#!/bin/bash -l

#PBS -q default

#PBS -l nodes=1:ppn=16

#PBS -l mem=64gb

#PBS -l walltime=4:00:00

#PBS -j oe

#PBS -A AG-Roth

export PATH=$HOME/bowtie2-2.0.0-beta6:$HOME/tophat-2.0.3.Linux_x86_64:$HOME/newtophat/samtools-0.1.18:$PATH

echo$PATH

#export NCORES=`cat $PBS_NODEFILE | wc -l`

#export OMP_NUM_THREADS=$NCORES

OUTDIR=/scratch/jlynch/zippednvngs

cd $OUTDIR

tophat2 -p 16 -r 0 -I 10000 -o 277TollA2610th20 -G /scratch/jlynch/zippednvngs/nv2clean.gff --no-novel-juncs ass1nvbowtie2 /scratch/jlynch/zippednvngs2/SN7640117_5879_TollA2610_1_sequence.fq.gz /scratch/jlynch/zippednvngs2/SN7640117_5879_TollA2610_2_sequence.fq.gz

**II. cufflinks commands annotation 2.0:**

247h2o805cl.ph

#!/bin/bash -l

#PBS -q default

#PBS -l nodes=1:ppn=16

#PBS -l mem=32gb

#PBS -l walltime=12:00:00

#PBS -j oe

#PBS -A AG-Roth

export PATH=$HOME/cufflinks-2.0.2.Linux_x86_64:$PATH

echo$PATH

#export NCORES=`cat $PBS_NODEFILE | wc -l`

#export OMP_NUM_THREADS=$NCORES

OUTDIR=/scratch/jlynch/zippednvngs

cd $OUTDIR

cufflinks -p 16 -o 247h2o805cl -G /scratch/jlynch/nvit2_evigenes_pub11u.goodclean.gff 197h2o805/accepted_hits.bam

247h2o1704cl.ph

#!/bin/bash -l

#PBS -q default

#PBS -l nodes=1:ppn=16

#PBS -l mem=32gb

#PBS -l walltime=12:00:00

#PBS -j oe

#PBS -A AG-Roth

export PATH=$HOME/cufflinks-2.0.2.Linux_x86_64:$PATH

echo$PATH

#export NCORES=`cat $PBS_NODEFILE | wc -l`

#export OMP_NUM_THREADS=$NCORES

OUTDIR=/scratch/jlynch/zippednvngs

cd $OUTDIR

cufflinks -p 16 -o 247h201704cl -G /scratch/jlynch/nvit2_evigenes_pub11u.goodclean.gff 197h2o1704/accepted_hits.bam

277dpp2610cl.ph

#!/bin/bash -l

#PBS -q default

#PBS -l nodes=1:ppn=16

#PBS -l mem=32gb

#PBS -l walltime=12:00:00

#PBS -j oe

#PBS -A AG-Roth

export PATH=$HOME/cufflinks-2.0.2.Linux_x86_64:$PATH

echo$PATH

#export NCORES=`cat $PBS_NODEFILE | wc -l`

#export OMP_NUM_THREADS=$NCORES

OUTDIR=/scratch/jlynch/zippednvngs

cd $OUTDIR

cufflinks -p 16 -o 277dpp2610cl -G /scratch/jlynch/nvit2_evigenes_pub11u.goodclean.gff 277dpp2610th20/accepted_hits.bam

277gbb1611cl.ph

#!/bin/bash -l

#PBS -q default

#PBS -l nodes=1:ppn=16

#PBS -l mem=32gb

#PBS -l walltime=12:00:00

#PBS -j oe

#PBS -A AG-Roth

export PATH=$HOME/cufflinks-2.0.2.Linux_x86_64:$PATH

echo$PATH

#export NCORES=`cat $PBS_NODEFILE | wc -l`

#export OMP_NUM_THREADS=$NCORES

OUTDIR=/scratch/jlynch/zippednvngs

cd $OUTDIR

cufflinks -p 16 -o 277gbb1611cl -G /scratch/jlynch/nvit2_evigenes_pub11u.goodclean.gff 277gbb1611th20/accepted_hits.bam

277TollA1611cl.ph

#!/bin/bash -l

#PBS -q default

#PBS -l nodes=1:ppn=16

#PBS -l mem=32gb

#PBS -l walltime=12:00:00

#PBS -j oe

#PBS -A AG-Roth

export PATH=$HOME/cufflinks-2.0.2.Linux_x86_64:$PATH

echo$PATH

#export NCORES=`cat $PBS_NODEFILE | wc -l`

#export OMP_NUM_THREADS=$NCORES

OUTDIR=/scratch/jlynch/zippednvngs

cd $OUTDIR

cufflinks -p 16 -o 277TollA1611cl -G /scratch/jlynch/nvit2_evigenes_pub11u.goodclean.gff 277TollA1611th20/accepted_hits.bam

277TollA2610cl.ph

#!/bin/bash -l

#PBS -q default

#PBS -l nodes=1:ppn=16

#PBS -l mem=32gb

#PBS -l walltime=12:00:00

#PBS -j oe

#PBS -A AG-Roth

export PATH=$HOME/cufflinks-2.0.2.Linux_x86_64:$PATH

echo$PATH

#export NCORES=`cat $PBS_NODEFILE | wc -l`

#export OMP_NUM_THREADS=$NCORES

OUTDIR=/scratch/jlynch/zippednvngs

cd $OUTDIR

cufflinks -p 16 -o 277TollA2610cl -G /scratch/jlynch/nvit2_evigenes_pub11u.goodclean.gff 277TollA2610th20/accepted_hits.bam

**III. cuffemerg commands annotation 2.0:**

cuffmerge -p 16 -o 277cuffmergeano20dwt -g /scratch/jlynch/nvit2_evigenes_pub11u.goodclean.gff -s assnv1.fa assembliesdwt.txt

**IV. cuffdiff commands annotation 2.0:**

#!/bin/bash -l

#PBS -q default

#PBS -l nodes=1:ppn=16

#PBS -l mem=64gb

#PBS -l walltime=16:00:00

#PBS -j oe

#PBS -A AG-Roth

export PATH=$HOME/cufflinks-2.0.2.Linux_x86_64:$PATH

echo$PATH

#export NCORES=`cat $PBS_NODEFILE | wc -l`

#export OMP_NUM_THREADS=$NCORES

OUTDIR=/scratch/jlynch/zippednvngs

cd $OUTDIR

cuffdiff -o 287mergedanno2ntnwnd -p 16 -L toll,wt,dpp -u 277cuffmergeano20dwt/merged.gtf /scratch/jlynch/zippednvngs/277TollA1611th20/accepted_hits.bam,/scratch/jlynch/zippednvngs/277TollA2610th20/accepted_hits.bam /scratch/jlynch/zippednvngs/197h2o805/accepted_hits.bam,/scratch/jlynch/zippednvngs/197h2o1704/accepted_hits.bam /scratch/jlynch/zippednvngs/277dpp2610th20/accepted_hits.bam,/scratch/jlynch/zippednvngs/277gbb1611th20/accepted_hits.bam

**V. Tophat commands annotation 1.2**

#!/bin/bash -l

#PBS -q default

#PBS -l nodes=1:ppn=16

#PBS -l mem=64gb

#PBS -l walltime=4:00:00

#PBS -j oe

#PBS -A AG-Roth

export PATH=$HOME/bowtie2-2.0.0-beta6:$HOME/tophat-2.0.3.Linux_x86_64:$HOME/newtophat/samtools-0.1.18:$PATH

echo$PATH

#export NCORES=`cat $PBS_NODEFILE | wc -l`

#export OMP_NUM_THREADS=$NCORES

OUTDIR=/scratch/jlynch/zippednvngs

cd $OUTDIR

tophat2 -p 16 -r 0 -I 10000 -o 257TollA2610an12 -G /scratch/jlynch/zippednvngs/1.2annot/ref_Nvit_2.0_scaffoldsedit.gff3 --no-novel-juncs nvitass2 /scratch/jlynch/zippednvngs2/SN7640117_5879_TollA2610_1_sequence.fq.gz /scratch/jlynch/zippednvngs2/SN7640117_5879_TollA2610_2_sequence.fq.gz

----------------------------------

#!/bin/bash -l

#PBS -q default

#PBS -l nodes=1:ppn=16

#PBS -l mem=64gb

#PBS -l walltime=4:00:00

#PBS -j oe

#PBS -A AG-Roth

export PATH=$HOME/bowtie2-2.0.0-beta6:$HOME/tophat-2.0.3.Linux_x86_64:$HOME/newtophat/samtools-0.1.18:$PATH

echo$PATH

#export NCORES=`cat $PBS_NODEFILE | wc -l`

#export OMP_NUM_THREADS=$NCORES

OUTDIR=/scratch/jlynch/zippednvngs

cd $OUTDIR

tophat2 -p 16 -r 0 -I 10000 -o 257TollA1611an12 -G /scratch/jlynch/zippednvngs/1.2annot/ref_Nvit_2.0_scaffoldsedit.gff3 --no-novel-juncs nvitass2 /scratch/jlynch/zippednvngs2/SN7640117_5882_TollA-1611_1_sequence.fq.gz /scratch/jlynch/zippednvngs2/SN7640117_5882_TollA-1611_2_sequence.fq.gz

----------------------------------

#!/bin/bash -l

#PBS -q default

#PBS -l nodes=1:ppn=16

#PBS -l mem=64gb

#PBS -l walltime=4:00:00

#PBS -j oe

#PBS -A AG-Roth

export PATH=$HOME/bowtie2-2.0.0-beta6:$HOME/tophat-2.0.3.Linux_x86_64:$HOME/newtophat/samtools-0.1.18:$PATH

echo$PATH

#export NCORES=`cat $PBS_NODEFILE | wc -l`

#export OMP_NUM_THREADS=$NCORES

OUTDIR=/scratch/jlynch/zippednvngs

cd $OUTDIR

tophat2 -p 16 -r 0 -I 10000 -o 257gbb1611an12 -G /scratch/jlynch/zippednvngs/1.2annot/ref_Nvit_2.0_scaffoldsedit.gff3 --no-novel-juncs nvitass2 /scratch/jlynch/zippednvngs2/SN7640117_5883_Gbb11611_1_sequence.fq.gz /scratch/jlynch/zippednvngs2/SN7640117_5883_Gbb11611_2_sequence.fq.gz

----------------------------------

#!/bin/bash -l

#PBS -q default

#PBS -l nodes=1:ppn=16

#PBS -l mem=64gb

#PBS -l walltime=4:00:00

#PBS -j oe

#PBS -A AG-Roth

export PATH=$HOME/bowtie2-2.0.0-beta6:$HOME/tophat-2.0.3.Linux_x86_64:$HOME/newtophat/samtools-0.1.18:$PATH

echo$PATH

#export NCORES=`cat $PBS_NODEFILE | wc -l`

#export OMP_NUM_THREADS=$NCORES

OUTDIR=/scratch/jlynch/zippednvngs

cd $OUTDIR

tophat2 -p 16 -r 0 -I 10000 -o 257dpp2610an12 -G /scratch/jlynch/zippednvngs/1.2annot/ref_Nvit_2.0_scaffoldsedit.gff3 --no-novel-juncs nvitass2 /scratch/jlynch/zippednvngs2/SN7640117_5880_dpp2610_1_sequence.fq.gz /scratch/jlynch/zippednvngs2/SN7640117_5880_dpp2610_2_sequence.fq.gz

-------------------------

#!/bin/bash -l

#PBS -q default

#PBS -l nodes=1:ppn=16

#PBS -l mem=64gb

#PBS -l walltime=4:00:00

#PBS -j oe

#PBS -A AG-Roth

export PATH=$HOME/bowtie2-2.0.0-beta6:$HOME/tophat-2.0.3.Linux_x86_64:$HOME/newtophat/samtools-0.1.18:$PATH

echo$PATH

#export NCORES=`cat $PBS_NODEFILE | wc -l`

#export OMP_NUM_THREADS=$NCORES

OUTDIR=/scratch/jlynch/zippednvngs

cd $OUTDIR

tophat2 -p 16 -r 0 -I 10000 -o 247h2o1704an12 -G /scratch/jlynch/zippednvngs/1.2annot/ref_Nvit_2.0_scaffoldsedit.gff3 --no-novel-juncs nvitass2 /scratch/jlynch/zippednvngs2/SN7640117_5878_H2O1704_1_sequence.fq.gz /scratch/jlynch/zippednvngs2/SN7640117_5878_H2O1704_2_sequence.fq.gz

-------------------------

#!/bin/bash -l

#PBS -q default

#PBS -l nodes=1:ppn=16

#PBS -l mem=64gb

#PBS -l walltime=4:00:00

#PBS -j oe

#PBS -A AG-Roth

export PATH=$HOME/bowtie2-2.0.0-beta6:$HOME/tophat-2.0.3.Linux_x86_64:$HOME/newtophat/samtools-0.1.18:$PATH

echo$PATH

#export NCORES=`cat $PBS_NODEFILE | wc -l`

#export OMP_NUM_THREADS=$NCORES

OUTDIR=/scratch/jlynch/zippednvngs

cd $OUTDIR

**VI. Cuffdiff assembly 1.2**

#!/bin/bash -l

#PBS -q default

#PBS -l nodes=1:ppn=16

#PBS -l mem=64gb

#PBS -l walltime=16:00:00

#PBS -j oe

#PBS -A AG-Roth

export PATH=$HOME/cufflinks-2.0.2.Linux_x86_64:$PATH

echo$PATH

#export NCORES=`cat $PBS_NODEFILE | wc -l`

#export OMP_NUM_THREADS=$NCORES

OUTDIR=/scratch/jlynch/zippednvngs

cd $OUTDIR

cuffdiff -o 267nmcd+banno12DvWvT -p 16 -L toll,wt,dpp -u /scratch/jlynch/zippednvngs/1.2annot/ref_Nvit_2.0_scaffoldsedit.gff3 -b /scratch/jlynch/zippednvngs/1.2annot/nvi_ref_Nvit_2.0concat4.fa 257TollA2610an12/accepted_hits.bam,257TollA1611an12/accepted_hits.bam 247h2o805an12/accepted_hits.bam,247h2o1704an12/accepted_hits.bam 257dpp2610an12/accepted_hits.bam,257gbb1611an12/accepted_hits.bam

tophat2 -p 16 -r 0 -I 10000 -o 247h2o805an12 -G /scratch/jlynch/zippednvngs/1.2annot/ref_Nvit_2.0_scaffoldsedit.gff3 --no-novel-juncs nvitass2 /scratch/jlynch/zippednvngs2/SN7640117_5881_H2O0805_1_sequence.fq.gz /scratch/jlynch/zippednvngs2/SN7640117_5881_H2O0805_2_sequence.fq.gz
